# Review of treatment modalities and clinical outcome of giant saccular posterior cerebral artery aneurysms

**DOI:** 10.1016/j.bas.2025.104309

**Published:** 2025-07-02

**Authors:** Andreas Theofanopoulos, Rajiv Kumar Khajuria, Dilaware Khan, Lucas Troude, Ben Waldau, Katharina Faust, Sajjad Muhammad

**Affiliations:** a Department of Neurosurgery, University Hospital of Patras, Patras, Greece; b Department of Neurosurgery, North University Hospital Marseille, APHM-AMU, Marseille, France; c Department of Neurological Surgery, UC Davis Medical Center, Sacramento, CA, USA; d Department of Neurosurgery, Medical Faculty and University Hospital Düsseldorf, Heinrich-Heine-Universität Düsseldorf, Düsseldorf, Germany

**Keywords:** Giant PCA aneurysm, Endovascular treatment, Surgical treatment, Outcome

## Abstract

**Introduction:**

Giant saccular posterior cerebral artery (PCA) aneurysms are rare lesions carrying significant morbidity due to mass effect and present therapeutic challenges, mainly due to the challenging approach required for aneurysm obliteration.

**Research question:**

To review treatment modalities and outcomes of patients harboring giant (>2.5 cm) PCA saccular aneurysms distal to the basilar bifurcation.

**Materials and methods:**

A systematic literature review through PubMed and Scopus to identify cases of giant saccular PCA aneurysms treated either microsurgically or endovascularly. Patients’ demographics, aneurysm size, preoperative and postoperative neurologic status, clinical outcomes and follow-up information were retrieved.

**Results:**

Data from 33 studies including 55 patients were obtained. Mean patient age was 34.35 years. Mean maximum aneurysm diameter was 38.48 mm. Presentation was aneurysm rupture in 30.9 %, headache in 23.6 %, hemiparesis or tetraparesis in 12.7 %, hemianopsia in 10.9 % and hydrocephalus in 5.5 %. At least 30.9 % had significant brainstem compression. Treatment was endovascular in 23.6 %, microsurgical in 67.3 % and combined in 9.1 %. Debulking to reduce mass effect was required in 32.4 %. Preoperative mRS ranged from 1 to 5. A favorable outcome (mRS 0–2) was reported on 92.7 % of cases. Death rate was 3.6 %. The PCA was sacrificed in 40 % of the patients without severe neurologic morbidity. Follow-up ranged from 1 week to 11 years.

**Discussion and conclusion:**

Giant PCA aneurysms are amenable to both treatment modalities. PCA sacrifice may be required and is often well tolerated, presumably due to the rich collateral supply. Mass effect may necessitate debulking. PCA bypass may be required, but carries significant morbidity.

## Highlights

• Giant saccular PCA aneurysms have malignant clinical courses and require treatment.
• Treatment requires complex endovascular and microsurgical techniques.
• Parent vessel sacrifice is often required and usually well tolerated.
• PCA sacrifice carries a risk of postoperative hemianopia.
• Brainstem mass effect may necessitate microsurgery for decompression.

## 1 Introduction

Saccular giant aneurysms of the posterior inferior cerebral artery (PCA) are rare but clinically important vascular lesions that present substantial therapeutic challenges. Defined as aneurysms with a diameter greater than 2.5 cm, saccular giant PCA aneurysms often arise near vital brainstem structures. They present with either mass effect on the brainstem or temporooccipital lobes with potential long tract findings or visual pathway dysfunction, cranial nerve dysfunction, obstructive hydrocephalus due to Sylvian aqueduct occlusion or transient ischemic attacks. Posterior circulation aneurysms also present a high risk of rupture and subsequent neurological deficits, urging for successful treatment (Wiebers, 2003). Treatment options have evolved over the years, with advances in both surgical and endovascular techniques offering potential solutions. The management strategy must consider aneurysm size, location, patient condition, and the risk of rupture, as well as the likelihood of preserving neurological function. Endovascular treatment is the first choice to treat small and medium size posterior circulation aneurysms but the optimal approach in giant saccular PCA aneurysms remains debatable (Molyneux et al., 2005). This review aims to provide a thorough review of treatment modalities for saccular giant PCA aneurysms and their outcomes.

## 2 Methods

The Preferred Reporting Items for Systematic Reviews and Meta-Analyses (PRISMA) Guidelines were used as a template for the methodology.

### 2.1 Search strategy

A comprehensive literature search was conducted through the PubMed and Scopus databases in February 2025, using the following terms: (PCA) OR (posterior cerebral artery), AND (aneurysm) AND (giant).

### 2.2 Inclusion and exclusion criteria

Descriptive and observational studies including case-control, longitudinal cohorts, cross-sectional studies, retrospective studies, review articles, editorials, commentaries, case series and case reports reporting outcomes of treatment of giant saccular aneurysms involving the PCA were included. Video articles were excluded. We excluded aneurysms' locations other than the PCA from our systematic review. Aneurysms of the PCA origin that included the basilar bifurcation were also excluded, as they were included in our group's already published systematic review of treatment modalities and outcomes of giant saccular aneurysms of the basilar apex (Theofanopoulos et al., 2024). Aneurysms with morphology described as fusiform or serpentine were also excluded. Studies not specifying treatment strategy, aneurysms' locations or clinical information about the patients were excluded. Studies where the size of the aneurysm was either not explicitly stated as >25 mm, or could not be confidently ascertained from provided imaging to be > 25 mm were also excluded. Finally, articles with full text in languages other than English were excluded.

### 2.3 Study selection

The duplicates from the initial database search were removed and a preliminary screening based on Title and Abstract was performed. Consequently, full-text screening was performed based on the predefined inclusion and exclusion criteria. Any conflicts were discussed and resolved by the senior author. The final included articles were reviewed and approved by all authors.

### 2.4 Data extraction

Reported follow-up were tabulated and reviewed. The modified Rankin Scale (mRS) was used as a measure of clinical outcome, with mRS scores of 0–2 denoting a ‘Favorable’ outcome and mRS 3–6 denoting a ‘Poor’ outcome for the subsequent analysis. In studies describing the patients' clinical status without specifying an mRS score, the score was calculated by the authors based on the clinical characteristics provided. In articles where the postoperative neurological status is described simply as improved, a mRS score of one point lower than the preoperative score was assumed. For studies providing the clinical outcome by measure of Glasgow Outcome Scale (GOS), the GOS score was converted to the corresponding mRS score based on the recommendations by Gaastra et al. (2022)

### 2.5 Statistical analysis

Comparison of variables was performed using the Chi-square (X^2^) test; Fisher's exact test was used instead if the expected frequency of at least an observation was less than 5. Missing observations were left blank during analysis. Statistical analysis was performed using IBM SPSS Statistics Version 28.0.2.0.

## 3 Results

**Fig. 1:**
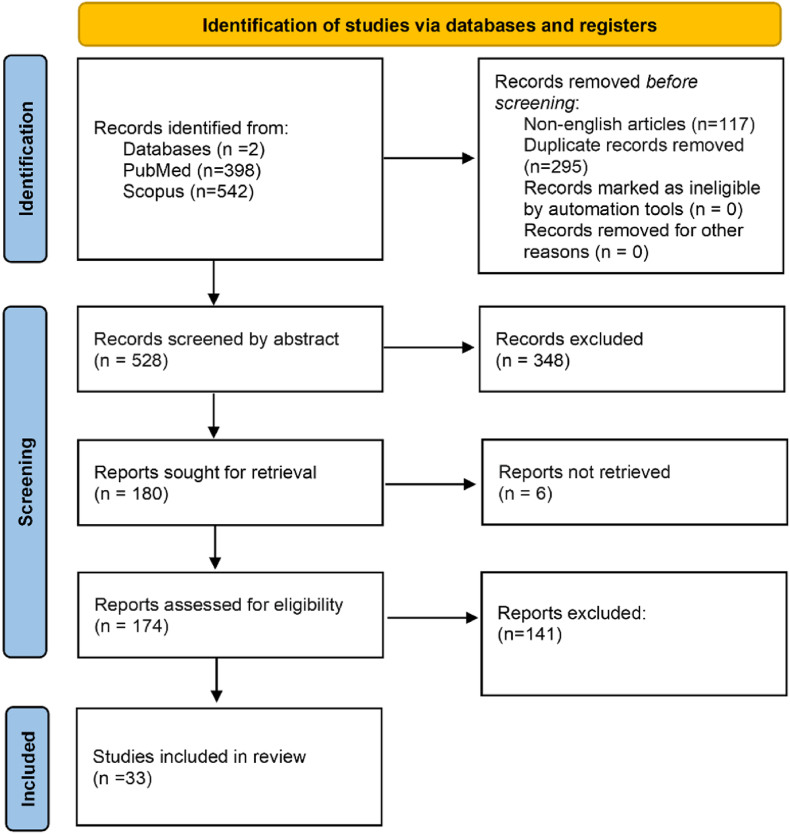
PRISMA flowchart.

The database search yielded a total of 940 studies. After removing articles written in languages other than English and removal of duplicates, 528 articles remained for abstract screening. Out of these, 174 articles remained for full-text screening. After exclusion of studies based on the predefined exclusion criteria, 33 studies were finally included in the review. The PRISMA flowchart is presented in Fig. 1.

The results are summarized in Table 1. There was a total of 55 patients reported in studies fulfilling the inclusion criteria.

**Table 1:** Giant saccular PCA aneurysms treated by open or endovascular techniques. M: male, F: female, SAH: subarachnoid hemorrhage, ICH: intracerebral hemorrhage, mRS: modified Rankin Scale, PCA: posterior cerebral artery, PCoA: posterior communicating artery, Mass: mass effect on brainstem, CN: cranial nerve, Bil: bilateral, ICA: internal carotid artery, Endo: endovascular, VP: ventriculoperitoneal, EVD: external ventricular drainage, EDH: epidural hematoma, E-E: end-to-end bypass, IMA: internal maxillary artery, RA: radial artery graft, E-S: end-to-side anastomosis, E-E: end-to-end anastomosis, NA: not available.

Study (Year)	N =	Age (Years)	Sex (M/F)	Size (Max diameter in mm)	Presentation	Anatomy	Previous treatment	Modality	Approach - Treatment	Additional procedures	Complication	preOP mRS	postOP mRS	Outcome	Follow-up (Months)
Obrador et al., 1967 (Obrador et al., 1967)	1	20	F	80	Vision loss	Fetal PCoA		Open	Transcortical - transoccipital - clipping - PCA sacrifice		Transient left hemiparesis	3	1	Favorable	0,5
Ventureyra et al., 1980 (Ventureyra et al., 1980)	1	0.5	F	90	Intracranial hypertension - hydrocephalus			Open	Subtemporal - clipping - reoperation for aneurysm excision - PCA sacrifice		Subdural effusion - subdural peritoneal shunt	4	1	Favorable	NA
Ley-Valle et al., 1983 (Ley-Valle et al., 1983)	1	9	M	26	Partial seizures			Open	Subtemporal - clipping- PCA sacrifice		Partial CN III palsy	1	0	Favorable	2
Meyer et al., 1989 (Meyer et al., 1989)	1	13	M	30	Headache			Open	Subtemporal - clipping			1	0	Favorable	NA
Scholten et al., 1992 (Scholten et al., 1992)	1	1	F	30	SAH			Open	Clipping			2	0	Favorable	NA
Yamashita et al., 1992 (Yamashita et al., 1992)	1	52	M	30	hemiparesis - ischemic stroke	P2		Endo	Balloon occlusion - PCA sacrifice			2	2	Favorable	1
Sakata et al., 1993 (Sakata et al., 1993)	1	14	M	30	Headache -hemianopsia	P2 - thrombosed		Open	Clipping - PCA sacrifice		Transient sensory aphasia	2	0	Favorable	5
Seoane et al., 1997 (Seoane et al., 1997)	4	30	M		Seizure	P1-2		Open	Pretemporal - trapping – PCA sacrifice + amygdalohippocampectomy			1	0	Favorable	NA
		47	F		Mass	P1-2		Open	Pretemporal - resection + E-E anastomosis			4	6	Unfavorable	NA
		41	M		SAH - hemianopsia	P2-3		Open	Subtemporal transventricular - clipping			2	2	Favorable	NA
		54	F		SAH	P2-3		Open	Subtemporal – trapping – PCA sacrifice			1	0	Favorable	NA
Arat et al., 2002 (Arat et al., 2002)	1	23	M		Headache	P2		Endo	Coiling - PCA sacrifice		Posterior thalamic infarct on MRI	1	0	Favorable	NA
Türe et al., 2003 (Türe et al., 2003)	1	37	M	50	Mass - hemiparesis	P2 - thrombosed - PCA thrombosed peripherally		Open	Pterional - excision - PCA sacrifice			2	2	Favorable	NA
Szajner et al., 2003 (Szajner et al., 2003)	1	64	F		Mass - hemiparesis	P1-2		Endo	Glue embolization - PCA sacrifice			2	0	Favorable	2,5
Hamada et al., 2005 (Hamada et al., 2005)	2	47	M		Mass	P2 - thrombosed		Open	Subtemporal – trapping – PCA sacrifice		Infarct - hemianopsia	1	2	Favorable	NA
		46	M	35	Hemianopsia	P2 - thrombosed		Open	Subtemporal - proximal clipping – PCA sacrifice		Transient sensory aphasia	2	0	Favorable	NA
Huang et al., 2005 (Huang et al., 2005)	1	14	F		Mass			Open	Clipping - debulking			2	1	Favorable	42
Biondi et al., 2006 (Biondi et al., 2006)	3	28	F		Headache	P2-3		Endo	PCA occlusion		Transient paresthesia and CN III paresis - posterior thalamic infarct	1	0	Favorable	66
		34	M		Headache	P2-3		Endo	PCA occlusion			1	0	Favorable	53
		25	F		SAH - hemiplegia	P2a		Endo	PCA occlusion		Hemianopsia - occipital infarct	4	2	Favorable	13
Li et al., 2008 (Li et al., 2008)	2	44	F		SAH - hemiparesis	P2		Endo	Coiling			4	2	Favorable	12
		10	M		Headache	P2		Endo	Coiling			1	0	Favorable	48
Vaid et al., 2008 (Vaid et al., 2008)	1	18	F		Mass - intracranial hypertension	P1-2		Open	Clipping			NA	2	Favorable	23
Poon et al., 2008 (Poon et al., 2008)	1	39	F		Headache - hemianopsia	P1-2		Endo	Coiling - PCA sacrifice		Lung atelectasia - pulmonary edema	2	2	Favorable	0.25
Waldron et al., 2009 (Waldron et al., 2009)	1	65	M	28	Mass		Coiling	Open	Clipping - debulking			1	1	Favorable	68.4
Bayrak et al., 2010 (Bayrak et al., 2010)	1	47	F	25	Headache - CN III palsy	P1-2 - PCoA supplying sac		Endo	Stent (balloon expandable) - PCoA outflow within sac coil embolized			2	1	Favorable	76
Chand et al., 2010 (Chang et al., 2010)	1	25	F		Headache - mass - hemiypesthesia - ataxia	P2-3 - fetal PCA		Combined	PCA bypass - coiling	EVD - EDH evacuation	Occipital infarct - EDH - sagittal sinus thrombosis	3	6	Unfavorable	NA
Zhitao et al., 2010 (Zhitao et al., 2010)	14	36	F		Mass	P2		Open	Subtemporal + pterional			NA	1	Favorable	NA
		38	M		SAH	P2		Open	Subtemporal + pterional			2	1	Favorable	NA
		55	F		Mass	P2 - thrombosed		Open	Subtemporal + pterional			NA	1	Favorable	NA
		33	M		Mass	P2 - thrombosed		Open	Subtemporal + pterional			NA	1	Favorable	NA
		21	F		SAH	P2 - AVM		Open	Subtemporal + pterional		Vasospasm	4	2	Favorable	NA
		37	F		Mass	P2		Open	Subtemporal + pterional			NA	1	Favorable	NA
		38	F		SAH	P2		Open	Subtemporal			NA	1	Favorable	NA
		29	F		SAH	P2 - thrombosed		Open	Subtemporal + pterional			NA	2	Favorable	NA
		44	M		Mass	P2 - thrombosed		Open	Subtemporal + pterional			NA	1	Favorable	NA
		41	F		SAH	P2		Open	Subtemporal			3	1	Favorable	NA
		31	F		SAH	P2 - thrombosed		Open	Subtemporal + pterional		Vasospasm	3	2	Favorable	NA
		42	F		SAH	P2		Open	Subtemporal + pterional			2	1	Favorable	NA
		53	F		SAH	P2		Open	Subtemporal + pterional		Vasospasm	3	2	Favorable	NA
		61	F		SAH	P2 - thrombosed		Open	Subtemporal + pterional		Vasospasm	3	2	Favorable	NA
Kim et al., 2013 (Kim et al., 2013)	3	18	F	27	Hemiparesis - CN III palsy	P1-2 - thrombosed		Open	Pretemporal - trapping – PCA sacrifice - debulking		Transient CN III palsy	3	1	Favorable	111.7
		36	M	30	ICH	P3-4		Open	Subtemporal - trapping PCA sacrifice - debulking		Transient CSF leak	NA	1	Favorable	77
		33	M	35	Hemianopsia	P1-2		Combined	Subtemporal + pretemporal - clipping + coiling		Transient hemiparesis - permanent hemianopsia	2	2	Favorable	15
Demartini et al., 2016 (Demartini et al., 2016)	1	10	M	27	SAH	P3	EVD	Endo	Coiling - PCA sacrifice			5	1	Favorable	12
Sarica et al., 2018 (Sarica et al., 2018)	1	10	F	35	Mass - hydrocephalus	P3	VP shunt	Endo	Coiling - PCA sacrifice	VP shunt removal		5	0	Favorable	72
Fukaya et al., 2017 (Fukaya et al., 2019)	1	78	M	40	Memory loss	P1-2 - thrombosed - Bil ICA occlusion - Klippel-Trenaunay syndrome		Open	Pterional - clipping			2	2	Favorable	NA
Horiuchi et al., 2020 (Horiuchi et al., 2020)	1	77	F	30	Mass - hydrocephalus	P3		Open	Occipital transtentorial - trapping – resection – PCA sacrifice			4	2	Favorable	3
Shimotsuma et al., 2020 (Shimotsuma et al., 2020)	1	17	F	33	Headache	P2-3		Endo	Stent assisted coiling	Recurrence - Coiling - PCA sacrifice		1	0	Favorable	3
Kadooka et al., 2020 (Kadooka et al., 2020)	1	54	M	31	Headache - ataxia - CN IV palsy	P1-2 - 2nd small P2 aneurysm - ICA atresia with supply from ipsilateral PCoA		Combined	STA-MCA bypass - stent assisted coiling		Venous midbrain + thalamus infarct	3	5	Unfavorable	24
O’Connor et al., 2020 (O'Connor et al., 2020)	1	5	M	50	Headaches	P2 - thrombosed		Combined	Coiling - PCA sacrifice - debulking		Transient hemiparesis - permanent quadrantanopsia	1	1	Favorable	3
SATO et al., 2021 (Sato et al., 2021)	1	62	F	30	Mass - hemiparesis	P2 temporal branch		Open	Subtemporal - trapping – PCA sacrifice - debulking			3	0	Favorable	2
O’Neal et al., 2021 (O'Neal et al., 2021)	1	7	M	68	Headaches - mental retardation			Combined	Coiling - PCA sacrifice - debulking		Quadrantanopsia	2	1	Favorable	9
Xu et al., 2021 (Xu et al., 2021)	1	14	F		SAH			Open	Clipping			NA	0	Favorable	135.8
Ding et al., 2024 (Ding et al., 2024)	1	62	F	33.5	Mass			Open	Zygomatic anterolateral temporal - IMA-RA-P2 E-S bypass - trapping - debulking	Reoperation - hematoma evacuation - bypass occlusion	Intraparenchymal hematoma	4	4	Unfavorable	7

### 3.1 Epidemiology

**Fig. 2:**
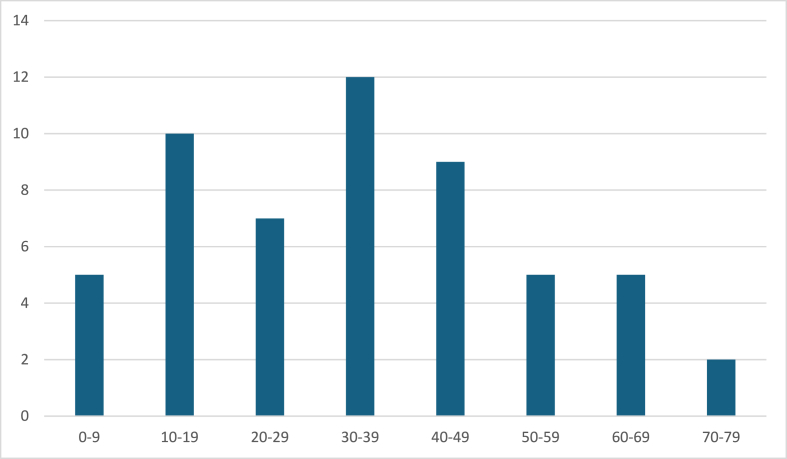
Distribution of the giant PCA aneurysms according to the patients' age at the time of treatment. Four out of five aneurysms were discovered before the 5th decade.

Almost four out of every five patients were before the 5th decade of their life, with one quarter being younger than 20 years old (Fig. 2). The mean patient age was 34.35 (0.5–78, SD 19.37) years, with a female preponderance of 58.2 %. Mean maximum aneurysm diameter was 38.48 (25–90, SD 17.29) mm.

### 3.2 Presentation

Subarachnoid or intracerebral hemorrhage was the presenting symptom in just 17/55 (30.9 %) of giant saccular PCA aneurysms. In at least another 17/55 (30.9 %) there was significant mass effect on the brainstem. Headache was the presenting symptom in 13/55 (23.6 %) of patients, while 3/55 (5.5 %) presented with cranial nerve dysfunction. Hemiparesis or tetraparesis was present in at least 7/55 (12.7 %) of patients and ataxia in 2/55 (3.6 %). A further 6/55 (10.9 %) presented with vision loss (hemianopsia), 3/55 (5.5 %) with obstructive hydrocephalus due to Sylvian aqueduct obstruction and 2/55 (3.6 %) presented with epileptic seizures. One patient (1.8 %) presented with memory loss and a child with inability to keep up with developmental milestones. In ten patients (18.2 %), the exact anatomic location of the aneurysm was not reported; of the rest, 10/45 (22.2 %) of the patients harbored aneurysms proximal to or at the P1-2 junction while the rest (77.8 %) involved more distal PCA segments.

### 3.3 Previous treatment

One patient (1.8 %) underwent ventriculoperitoneal shunting prior to aneurysm treatment due to obstructive hydrocephalus caused by mass effect on the Sylvian aqueduct, while another one needed external ventricular drainage or ventriculoperitoneal shunt due to hydrocephalus caused by SAH.

In one patient, a history of previous unsuccessful treatment of the same PCA aneurysm was reported. This was a patient who was initially treated with endovascular coiling, where angiographic recurrence in the form of coil compaction led to a decision for microvascular clipping and aneurysm debulking by thrombectomy to relieve mass effect.

### 3.4 Treatment modalities

Out of 55 patients, 13 (23.6 %) received endovascular treatment, while 37 (67.3 %) underwent microsurgical treatment. Five patients (9.1 %) underwent hybrid treatment with endovascular trapping of the aneurysm and subsequent microsurgical debulking. Aneurysm rupture at admission was present in 14 (37.8 %) of the surgically treated patients and 3 (23.1 %) of the ones who received endovascular treatment. At least 12/55 (21.8 %) of the aneurysms were partially thrombosed, only one of which received combined treatment with endovascular PCA occlusion and microsurgical aneurysm debulking; the rest were treated microsurgically.

The most commonly utilized endovascular procedure was simple aneurysm coiling, which was used in 46.2 % of the endovascularly treated patients. More complex approaches included stent-assisted coiling and balloon expandable stenting, while earlier approaches included detachable balloon embolization or glue embolization (in both cases with parent artery sacrifice). PCA preservation with simple coiling of the aneurysm was possible in only 2 (15.4 %) of the endovascular cases, with another 2 cases requiring complex techniques (stenting and stent-assisted coiling). Endovascular treatment included PCA occlusion/sacrifice as part of the strategy for aneurysm occlusion in 9 cases (69.2 %).

Microsurgical treatment strategy consisted of either direct aneurysm clipping or aneurysm excision or trapping with peripheral PCA sacrifice. The latter was required in at least 13 patients (35.1 %). The most commonly utilized approach was the subtemporal one, which was used alone in at least 12/37 (32.4 %) and combined with a pterional approach in at least 24/37 (64.9 %) of the cases. A pterional approach with extension to the floor of the middle fossa and complete exposure of the temporal lobe is termed the pretemporal approach (also known as the ‘half and half’ approach). It allows for a wide Sylvian fissure split and a wide opening of the basal cisterns and can provide access to the P1 and P2 segments of the PCA. It also allows for a subtemporal view and therefore may offer a more complete exposure of the giant aneurysm's anatomy, which may be the reason for its popularity among the reports included in the present study.

Five patients were treated with strategies combining endovascular interventions and microsurgical approaches. In one case, coiling was utilized to completely secure the aneurysm after intraoperative aneurysmal rupture during microsurgery and partial aneurysm clipping. In two cases, microsurgical debulking was required after securing the aneurysm by endovascular coiling. In a further two cases, a bypass procedure was utilized before endovascular aneurysm obliteration; in one case to secure distal PCA perfusion, in another case a STA-MCA bypass to secure adequate perfusion of the anterior circulation in case of PCoA compromise due to ICA atresia before stent assisted coiling of the giant aneurysm.

A bypass for reconstitution of PCA territory supply was performed in 3 cases (8.1 % of the total patients). These consisted of an only graft, a prophylactic STA-MCA bypass due to anterior circulation supply by the posterior circulation through the posterior communicating artery, and an internal maxillary artery to P2 end-to-side bypass with a radial artery interposition graft. (Chang et al., 2010), (Kadooka et al., 2020), (Ding et al., 2024) In one case, aneurysm excision and end-to-side PCA anastomosis was performed via the pretemporal route (Seoane et al., 1997).

Aneurysmectomy or aneurysm debulking was performed in 12 cases (32.4 %).

In total, parent vessel sacrifice was required in at least 22/55 (40 %) of cases and was not associated with a poor outcome, with all patients achieving a favorable outcome. Despite a favorable outcome, hemianopsia or other visual deficits were reported in 5 (9.8 %) of the total cases. A definite ischemic event attributable to the parent vessel occlusion and certified by postoperative imaging or an unequivocal clinical picture was reported in 7/22 (31.8 %) of the patients that underwent PCA sacrifice.

### 3.5 Outcomes

Postoperative angiographic results were only reported in some of the endovascularly treated cases; therefore this parameter was not included in the present study.

A favorable outcome (mRS 0–2) was reported on 92.7 % of cases. Death rate was 3.6 %. The parent vessel was sacrificed in about 22/55 (40 %) of the patients without severe neurologic morbidity. Of those patients, 9/22 (40.9 %) were reported to either have a radiologically confirmed infarct or neurological sequelae compatible with infarction from PCA occlusion, however all patients achieved a favorable outcome (mRS 0–2). Only three of those patients (13.6 % of the total parent vessel sacrifices) had complete hemianopsia.

An unfavorable outcome (mRS >2) was reported in four cases. A patient with severe brainstem compression preoperatively that underwent aneurysm trapping and excision with end-to-end PCA anastomosis eventually succumbed to unspecified postoperative complications (Seoane et al., 1997). One bypass case involved a postoperative intraparenchymal hematoma (Ding et al., 2024). One combined case developed a postoperative occipital infarct as well as an EDH compressing and thrombosing the superior sagittal sinus after craniotomy and PCA bypass (Chang et al., 2010). Another combined case developed a venous infarct of the midbrain and thalamus because of post-interventional aneurysm size increase, compromising the venous drainage of the mesencephalon (Kadooka et al., 2020). No endovascular only treated patient suffered an unfavorable outcome.

Patients requiring vessel anastomosis seem to have a significant risk of an unfavorable outcome (p < 0.001) or death (p = 0.004). Combination of microsurgical and endovascular techniques seems to carry a significant association with unfavorable outcomes (p = 0.037); however this could be influenced by the possibility that cases treated with hybrid techniques may represent more complex cases to begin with. In cases where a single modality was used, there was no significant difference in the outcomes or deaths in the present study.

Subarachnoid hemorrhage was not significantly associated with outcomes in the present study (p = 0.299).

There was one reported recurrence during the follow-up periods. This was a patient that initially underwent a stent-assisted coiling of a P2-3 aneurysm, which recurred within 3 months and was definitively treated by endovascular parent artery occlusion (Shimotsuma et al., 2020).

## 4 Discussion

Giant saccular aneurysms of the posterior cerebral artery most often present before the age of 50 years, in contrast to the epidemiology of cerebral aneurysms in general, which tend to be discovered around the 60th year of life, while the female preponderance is consistent with the literature (Zuurbier et al., 2022).

Due to their location, giant PCA aneurysms usually become symptomatic with hemiparesis, hemihypesthesia or ataxia, due to midbrain and cerebral peduncle compression. Another possible presenting phenotype is obstructive hydrocephalus, due to Sylvian aqueduct stenosis and/or eventual occlusion. Cranial nerve dysfunction (oculomotor and/or trochlear) was also a common symptom, either pre- or postoperatively. Hemianopsia is another common presentation, possibly due to ischemic events. Hemorrhage occurred in about one third of patients and was not significantly associated with worse outcomes in the present study, possibly due to the equally severe presentation of the patients presenting with mass effect.

The surgical modalities employed consisted of clip occlusion, parent artery proximal occlusion, or aneurysm trapping. Excision or debulking of the aneurysm was performed in one third of the patients to relieve the mass effect associated with the aneurysm. The most often used approaches were the subtemporal and the pterional or its variants, or in some cases the combination of both to offer wider exposure.

Endovascular treatment consisted either of simple aneurysm coiling or balloon occlusion in earlier series, stent-assisted aneurysm coiling, or parent artery occlusion with coils or glue.

While microsurgical treatment of PCA aneurysms is not straightforward due to the deep and narrow working corridors and proximity to brainstem and cranial nerves, endovascular treatment is also not without its respective challenges.

This is in part due to inconvenient geometry of the angle of P2 branches' origin and the vessel's small caliber, which can make catheterization difficult.

The results of the present study suggest that treatment of such aneurysms often necessitates parent vessel sacrifice. This seems to be well tolerated by the majority of the patients, with the major clinical complication being quadrantanopia or hemianopia. This complication however seems to be less common one could anticipate, probably due to the rich leptomeningeal anastomotic network between distal PCA territories and anterior cerebral and middle cerebral artery branches (Hallacq et al., 2002). On the other hand, bypass of the aneurysm may carry significant morbidity, as suggested also in the present study. Therefore, careful patient selection and counselling should be utilized, taking into account that parent vessel occlusion may be tolerated with no or acceptable deficits; the risk of hemianopia should be weighed against this of the bypass morbidity (Chang et al., 2010). Preoperative balloon test occlusion was not routinely utilized in most studies, and no clear guidelines can be drawn as to when it should be implemented – its inclusion may be reasonable in patients that have intact vision and who deem a postoperative visual field deficit as unacceptable, where parent vessel sacrifice is deemed unavoidable and a revascularization procedure is estimated as technically feasible with low morbidity by the treating team.

Hybrid treatment, with endovascular aneurysm and parent vessel occlusion followed by microsurgical aneurysm decompression by debulking, may be needed in cases of severe symptomatic brainstem or cranial nerve compression. However, the additive morbidity of both operations may contribute to a higher probability of an unfavorable outcome. Therefore, combination of treatment modalities should be used with caution and reserved for patients where either endovascular or microsurgical access cannot in itself safely provide definitive treatment.

Due to the fact that most cases had a favorable outcome regardless of the treatment modality, no comparison can be drawn between the two treatment modalities.

Furthermore, the mass effect associated with giant PICA aneurysms, which are often partially thrombosed, is a major factor causing symptomatology and may often not be alleviated by endovascular means. In such cases, the necessity to debulk the aneurysm by thrombus removal or even complete aneurysmectomy may call for open microsurgical approaches in an effort to offer definitive cure.

### 4.1 Limitations

A significant limitation of the present study is the relatively small sample size, which does not lend itself to safe clinical conclusions about treatment options. Another possible limitation may be selection bias, as in cases that may have been amenable to both treatment modalities, the treating teams may have opted for the modality most favorable for each particular case or the one that was most readily available. Furthermore, the study includes cases that were treated before the introduction of endovascular techniques; as a result the percentage of cases treated microsurgically may be artificially higher than what the current situation would dictate. A further limitation is the widely variable follow-up length between the studies, ranging from 1 week to more than 11 years, as well as the omission of follow-up duration data in many of the studies.

Some series reporting treatment of PCA aneurysms were also omitted due to not providing explicit data on the clinical course of individual patients or the size, location and morphology of the aneurysms. However, this may skew the analysis by excluding some experienced surgeons’ results.

Another limitation is that not all patients may have undergone detailed neuro-ophthalmological evaluations to rule out visual field deficits after PCA sacrifice, or visual field deficits may not have been reported due to recall bias. The primary outcome in this study was modified Rankin scale score 0–2, however, patients with hemianopia can also reach this favorable outcome score even if they may not be able to drive due to their visual field deficit.

Even considering the above limitations, the study provides a useful overview of treatment outcomes of the most popular treatment modalities for giant PCA aneurysms and can serve as a useful reference frame for clinical decision-making.

## 5 Conclusion

Giant saccular PCA aneurysms are amenable to treatment with either endovascular or microsurgical techniques. Parent vessel sacrifice is often required, especially in endovascular techniques; this is usually well tolerated, probably due to the rich leptomeningeal anastomotic supply and potentially due to a windkessel effect of the giant aneurysm which allows for collaterals to develop. Nevertheless, patients undergoing PCA vessel sacrifice should be counselled about the risk of postoperative hemianopia. Therefore, careful patient selection and counselling guide the decision for revascularization of the distal PCA territory, as bypass seems to carry significant morbidity. Due to the small sample size, as well as lesion and treatment modality heterogeneity in the available literature, solid conclusions regarding choice of treatment cannot be drawn. Brainstem compression may necessitate microsurgical aneurysm decompression or removal, with a high probability of a favorable outcome. Ideally, decision-making should be individualized in an interdisciplinary setting with both endovascular and microsurgical capabilities.

## Declaration of competing interest

The authors declare that they have no known competing financial interests or personal relationships that could have appeared to influence the work reported in this paper.
